# The transcription factor STAT6 plays a critical role in promoting beta cell viability and is depleted in islets of individuals with type 1 diabetes

**DOI:** 10.1007/s00125-018-4750-8

**Published:** 2018-10-18

**Authors:** Kaiyven A. Leslie, Mark A. Russell, Kazuto Taniguchi, Sarah J. Richardson, Noel G. Morgan

**Affiliations:** Institute of Biomedical and Clinical Sciences, University of Exeter Medical School, RILD Building (Level 4), Barrack Road, Exeter, EX2 5DW UK

**Keywords:** Cytokine, Inflammation, Interleukin-4, Interleukin-13, Palmitate, SIRPα

## Abstract

**Aims/hypothesis:**

In type 1 diabetes, selective beta cell loss occurs within the inflamed milieu of insulitic islets. This milieu is generated via the enhanced secretion of proinflammatory cytokines and by the loss of anti-inflammatory molecules such as IL-4 and IL-13. While the actions of proinflammatory cytokines have been well-studied in beta cells, the effects of their anti-inflammatory counterparts have received relatively little attention and we have addressed this.

**Methods:**

Clonal beta cells, isolated human islets and pancreas sections from control individuals and those with type 1 diabetes were employed. Gene expression was measured using targeted gene arrays and by quantitative RT-PCR. Protein expression was monitored in cell extracts by western blotting and in tissue sections by immunocytochemistry. Target proteins were knocked down selectively with interference RNA.

**Results:**

Cytoprotection achieved with IL-4 and IL-13 is mediated by the early activation of signal transducer and activator of transcription 6 (STAT6) in beta cells, leading to the upregulation of anti-apoptotic proteins, including myeloid leukaemia-1 (MCL-1) and B cell lymphoma-extra large (BCLXL). We also report the induction of signal regulatory protein-α (SIRPα), and find that knockdown of SIRPα is associated with reduced beta cell viability. These anti-apoptotic proteins and their attendant cytoprotective effects are lost following siRNA-mediated knockdown of STAT6 in beta cells. Importantly, analysis of human pancreas sections revealed that STAT6 is markedly depleted in the beta cells of individuals with type 1 diabetes, implying the loss of cytoprotective responses.

**Conclusions/interpretation:**

Selective loss of STAT6 may contribute to beta cell demise during the progression of type 1 diabetes.

**Electronic supplementary material:**

The online version of this article (10.1007/s00125-018-4750-8) contains peer-reviewed but unedited supplementary material, which is available to authorised users.

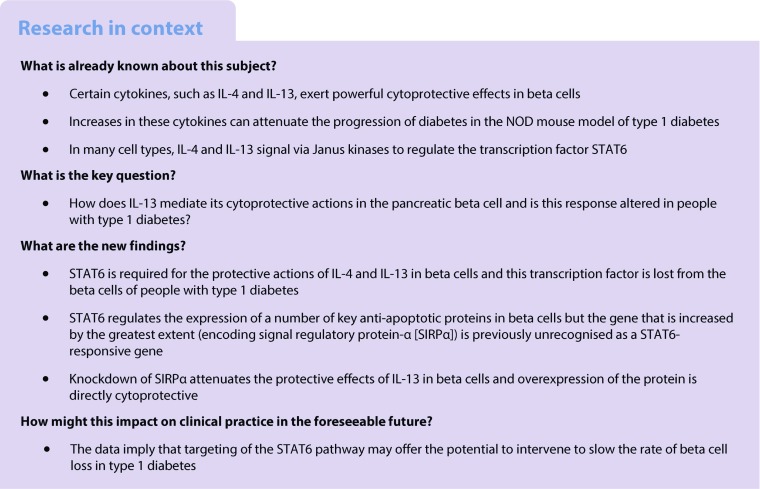



## Introduction

Type 1 diabetes arises following the selective destruction of pancreatic beta cells by immune cells that infiltrate the islets of Langerhans during disease development. In humans, these infiltrates contain various different immune cell subtypes but are dominated by autoreactive CD8^+^ T cells [1–3], considered to be the principal mediators of beta cell demise [4]. Loss of beta cells occurs via a combination of direct cell-mediated toxicity and the release of soluble factors (such as proinflammatory cytokines, granzymes and perforin) which promote apoptosis [5–9]. However, the situation is complex since balanced against these pro-apoptotic mechanisms favouring beta cell loss are factors which act to sustain viability, including anti-inflammatory cytokines such as IL-13 and IL-4. In common with their proinflammatory counterparts, these may be secreted from specific immune cell subsets [10–14] and might also originate from the islet cells. Irrespective of their precise source, the final outcome of any inflammatory episode for beta cell viability will be determined by the competing effects of these various antagonistic influences operating within the islet milieu.

The actions of proinflammatory cytokines have been well-studied in beta cells [9, 12, 15–18] but, in contrast, the counter-balancing effects of anti-inflammatory molecules have received much less attention. Nevertheless, it is known that exogenous administration of IL-13 or IL-4 reduces the incidence and delays the onset of diabetes in the NOD mouse model of type 1 diabetes [10, 19] and that IL-4 and IL-13 each exert direct pro-survival effects in human pancreatic islet cells [20–22]. Thus, it is likely that the availability of such molecules within islets may influence the survival of beta cells in the face of ongoing autoimmunity.

IL-4 and IL-13 share approximately 30% sequence homology [23] and both interact with cell surface receptors containing the ‘IL-4RA’ subunit. In the case of IL-4, this subunit is complexed with the common γ-chain to form the functional receptor, whereas in the IL-13 receptor, IL-4RA interacts with IL-13Rα1 [24]. All of these components are expressed in human islets and on clonal beta cell lines [25, 26]. Upon binding of their cognate cytokines, each receptor promotes the auto-phosphorylation of associated Janus kinases (JAKs), leading to a cascade of events culminating in the recruitment and phosphorylation of the transcription factor, signal transducer and activator of transcription 6 (STAT6). In response, STAT6 monomers dimerise and translocate to the nucleus where they bind to consensus sequences in genomic DNA to promote the transcription of target genes [27]. Previous studies have confirmed that IL-13 treatment induces a robust and early phosphorylation of STAT6 in the beta cell, in a JAK-dependent manner, demonstrating that this pathway is operational in these cells [20].

Despite this evidence, the mechanisms involved in promoting beta cell viability in response to STAT6 activation are unclear and it is not known whether this pathway is altered during the autoimmune attack associated with type 1 diabetes. Therefore, to address these issues, we have disrupted STAT6 signalling specifically in clonal beta cells and studied its effects on the actions of IL-4 and IL-13.

## Methods

### Imaging studies

Human formalin-fixed paraffin-embedded (FFPE) pancreas samples were obtained from the Exeter Archival Diabetes Biobank (http://foulis.vub.ac.be/), an archival collection of post-mortem pancreas samples [28]. These consisted of six samples taken from individuals with recent-onset type 1 diabetes plus six control samples from individuals of similar age and sex (ESM Table 1). All samples were studied with full ethical approval (15/W/0258).

Where a single antigen was examined, sections were studied using immunoperoxidase staining. In these experiments, samples were dewaxed, rehydrated and heated in a citrate antigen retrieval buffer (pH 7.4) for 20 min using a microwave (800 W). Sections were blocked in 5% (vol./vol.) normal goat serum and then probed with primary and relevant secondary antibodies (ESM Table 2). Haematoxylin was used as a counterstain prior to mounting of the section in distyrene–xylene-based mountant. Alternatively, where two or more antigens were examined, sections were sequentially stained with primary antibodies and species-appropriate fluorescently labelled secondary antisera prior to mounting. Antisera were validated either using relevant control tissues or were used in accordance with manufacturers’ instructions.

Images were captured using an AF6000 fluorescent microscope (Leica Microsystems, Milton Keynes, UK). For quantification studies, randomly selected insulin-containing islets from individuals with and without diabetes were imaged using identical microscope and camera settings. Regions of interest were drawn around the islet periphery and the mean fluorescence intensity (MFI) of each antigen was assessed using Image J version 1.50b (https://imagej.nih.gov/ij/) with Java 1.8.0_77 https://www.oracle.com/technetwork/java/javase/8u77-relnotes-2944725.html). Alternatively, the MFI of STAT6 immunostaining was calculated only in insulin-positive regions using a custom MATLAB script (version R2015b) (www.mathworks.com/products/new_products/release2015b.html), VIOLA, developed in house (University of Exeter). The VIOLA script identifies insulin-positive regions within each image by thresholding against insulin-negative regions. It then reports the MFI of a second antigen (in this case STAT6) only in areas where the two are co-localised.

### Cell culture and treatments

Cultured INS-1E cells (a gift from C. Wollheim, University of Geneva, Switzerland) were used to perform most in vitro experiments. Cell culture was achieved in RPMI-1640 at 11 mmol/l glucose (Lonza, Basel, Switzerland) supplemented with 10% FBS, 2 mmol/l l-glutamine, 100 U/ml penicillin, 100 μg/ml streptomycin and 50 mmol/l 2-mercaptoethanol (all from ThermoFisher, Boston, MA, USA) and treatments were performed at 2 × 10^5^ cell/ml seeding density [29]. Cells were treated with 20 ng/ml of each cytokine (IL-4, IL-13, IL-1β, TNF-α, IFN-γ and IL-6; all from R&D systems, Abingdon, UK), 250 μmol/l palmitic acid complexed to 1% bovine serum albumin or after serum withdrawal for the desired duration. These concentrations were chosen since they had been shown to be effective in our previous studies [20, 30]. Human EndoC-βH1 beta cells were sourced and cultured as described in [31]. Cells were routinely tested for mycoplasma contamination and were negative.

### Knockdown of STAT6 and SIRPα

Knockdown of target transcripts was achieved using small interference RNAs (siRNAs) for rat *Stat6* and *Sirpα* (also known as *Sirpa*) (ThermoFisher). Cells were transfected with target or scrambled siRNA. Scrambled siRNA was generated randomly from the *Stat6* sequence (GAAUUAAUCGUCGUCUU), and tested against the NCBI database to confirm the lack of off-target effects. Commercial siRNA sequences are proprietary. Optimem (ThermoFisher) and lipofectamine RNAi Max (Invitrogen, Boston, MA, USA) were used as transfection reagents and successful knockdown was confirmed by western blotting and/or quantitative reverse transcription PCR (qRT-PCR).

## Overexpression of SIRPα

SIRPα was overexpressed in INS-1E cells using a pCMV6 vector containing the *SIRPΑ* coding sequence (Origene, Rockville, MD, USA). Transfection of this construct or an empty vector was performed using Lipofectamine LTX reagent (Invitrogen) 24 h prior to each experiment. Transfection was confirmed by western blotting and/or qRT-PCR.

### Western blotting

Cellular proteins were extracted and used for western blotting as previously described [20]. Primary antibodies (ESM Table 2) were added at 4°C in blocking solution unless stated otherwise. After overnight incubation, membranes were washed for 15 min in tris-buffered saline–Tween (TBST) and probed with appropriate alkaline phosphatase-conjugated secondary antibodies (Merck, Darmstadt, Germany) for 1 h at room temperature. Bands were detected with CDP-star chemiluminescent substrate (Merck) or by Licor Odyssey detection system (Licor, Cambridge, UK) when fluorescent secondary antibodies were used. Densitometric analysis was performed using Image Studio version 5.2 (https://www.licor.com/bio/products/software/image_studio/) after normalising for expression of β-actin or glyceraldehyde 3-phosphate dehydrogenase (GAPDH).

### qRT-PCR

RNA was extracted from cells using an RNeasy Mini kit (Qiagen, Hilden, Germany) and its quantity and quality were estimated by NanoDrop measurement (ThermoFisher). RNA (500 ng) was used for cDNA synthesis (Qiagen) and gene expression was monitored by qRT-PCR with SYBR Green master mix using commercially available RT2 Profiler PCR Array and primers for genes of interest (Qiagen). Amplicons were generated on the QuantStudio Flex 12K (Applied Biosystems, Boston, MA, USA) and gene expression was calculated using the comparative threshold cycle method (${2}^{-\Delta \Delta {\mathrm{C}}_{\mathrm{t}}}$) after normalising with transcripts encoding *Hprt1* and *Yy1* [32].

### Cell viability measurements

Viability was estimated using either Trypan Blue (0.4% wt/vol. in PBS) or propidium iodide (PI) (Merck) as previously described [26]. Routinely, each experimental condition was replicated six times and individual experiments were repeated on at least three separate occasions.

### Cell cycle analysis by flow cytometry

A single time point cell cycle analysis was performed by PI staining as described [33].

### Statistics

All statistical analyses were performed on Graphpad Prism version 7.0 (https://www.graphpad.com/scientific-software/prism/) and data are presented as mean values ± SEM. Unpaired Student’s *t* test or ANOVA (with post hoc Tukey’s test) were used to assess statistical significance between mean values. Data were considered statistically significant when *p* < 0.05.

## Results

### IL-13 and IL-4 each induce STAT6 phosphorylation and cytoprotection in cultured beta cells

**Fig. 1:**
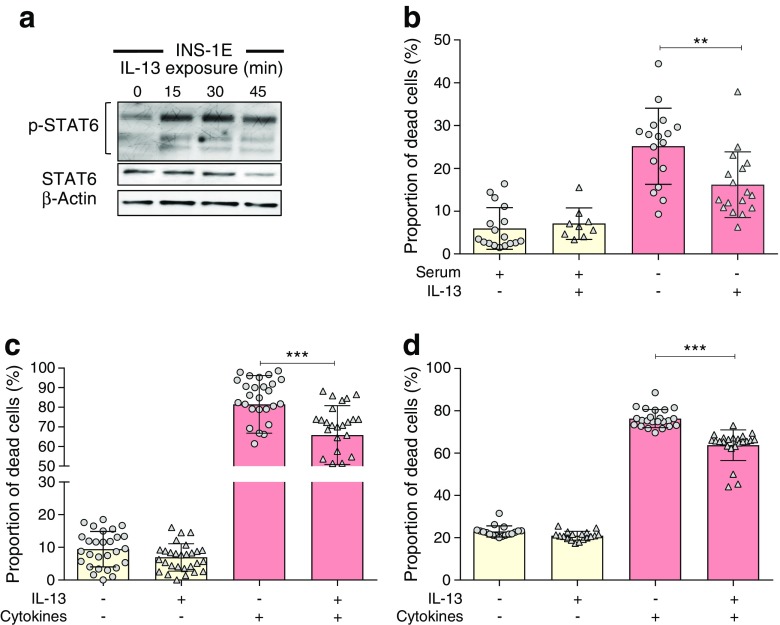
STAT6 is phosphorylated in response to IL-13 and protects beta cells from the effects of serum withdrawal or addition of proinflammatory cytokines. (**a**) INS-1E cells were treated with IL-13 (20 ng/ml) for 45 min. The cells were lysed and protein extracted for western blotting to detect p-STAT6, total STAT6 and β-actin. (**b**–**d**) INS-1E cells (**b**, **c**) or EndoC-βH1 cells (**d**) were treated with IL-13 (20 ng/ml) for either 96 h in the absence of serum (**b**) or for 48 h in the presence of a proinflammatory cytokine cocktail (20 ng/ml of IL-1β, TNFα, IFNγ and IL-6) (**c**, **d**). Cell viability was determined either by Trypan Blue (**b**, **c**) or PI staining (**d**). All data represent mean values from three independent experiments ± SEM. **p<0.01 and ***p<0.001 as indicated

The cytoprotective actions of IL-13 were investigated in rodent INS-1E and human EndoC-βH1 cells. As expected, withdrawal of serum or treatment of INS-1E cells with a proinflammatory cytokine cocktail caused a dramatic loss of viability, which was attenuated by IL-13 (Fig. 1b–c). IL-4 also protected beta cells against serum withdrawal (serum withdrawal 53.5 ± 1.5% cell death; serum withdrawal + IL-4 44.4 ± 1.1%; *p* < 0.01). Importantly, an equivalent cytoprotective response to IL-13 was also observed in EndoC-βH1 cells treated with proinflammatory cytokines (Fig. 1d), confirming that the response is not restricted only to rodent cells. IL-13 treatment of INS-1E cells led to increased tyrosine phosphorylation of STAT6 (Fig. 1a) within 15 min and, as seen previously [20], three separate immunoreactive pSTAT6 bands could be detected by western blotting, all of which were enhanced in response to IL-13.

### Silencing of STAT6 abrogates the cytoprotective effect of IL-13

**Fig. 2:**
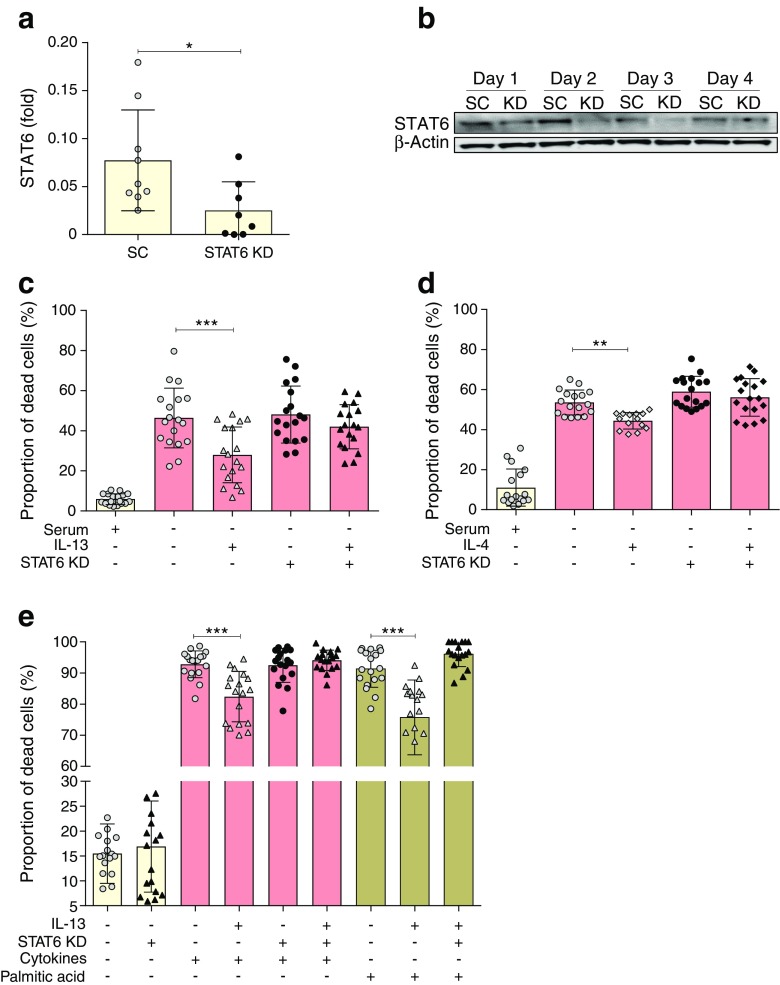
Silencing of *Stat6* abrogates the cytoprotective effects of IL13. (**a**, **b**) INS-1E cells were transfected with siRNA targeting *Stat6* (knockdown [KD]) or with a scrambled control siRNA (SC), and incubated for up to 96 h. Cell lysates were extracted and western blotting performed. Membranes were probed with antisera recognising STAT6 and β-actin. Expression of STAT6 was quantified after 48 h knockdown by densitometric analysis, with data expressed relative to β-actin (*n*=3). (**c**–**e**) STAT6 expression was depleted with siRNA for 24 h prior to addition of IL-13 (20 ng/ml) (**c**, **e**) or IL-4 (20 ng/ml) (**d**). Cells were incubated in the absence of serum for 96 h (**c**, **d**) or in the presence of a proinflammatory cytokine cocktail (20 ng/ml of IL-1β, TNFα, IFNγ and IL-6) or 250 μmol/l palmitate, as shown, for 48 h (**e**). Cells were harvested and viability was assessed by Trypan Blue staining. Data represent mean values from three independent experiments ± SEM. *p<0.05, **p<0.01 and ***p<0.001 as indicated

To understand the importance of STAT6 in mediating the cytoprotective response to IL-13 in beta cells, siRNA molecules targeting *Stat6* selectively were employed. Transfection of *Stat6* siRNA into INS-1E cells caused an approximately 75% reduction in STAT6 protein levels relative to the scrambled siRNA-treated control cells, within 48 h (Fig. 2a). STAT6 knockdown was stable for at least 4 days (Fig. 2b) but returned to pre-treatment levels within 6 days of transfection (not shown).

To examine the effect of depletion of STAT6 on the ability of IL-13 to promote cytoprotection, siRNA directed against *Stat6* was transfected into cells for a period of 24 h prior to treatment with IL-13. The cells were then exposed either to a period of serum withdrawal or were treated with a cocktail of proinflammatory cytokines or 250 μmol/l palmitate. As expected, IL-13 improved the viability of cells incubated under each of these conditions (Fig. 2c, e). By contrast, when STAT6 was knocked down prior to IL-13 treatment, the cytoprotective responses were abrogated. The protective action of IL-4 was similarly sensitive to STAT6 knockdown (Fig. 2d). Interestingly, however, knockdown of STAT6 did not itself lead to any loss of beta cell viability in the absence of a specific cytotoxic stimulus.

### Anti-apoptotic and anti-inflammatory genes are upregulated following IL-13 treatment of INS-1 cells in a STAT6-dependent manner

**Fig. 3:**
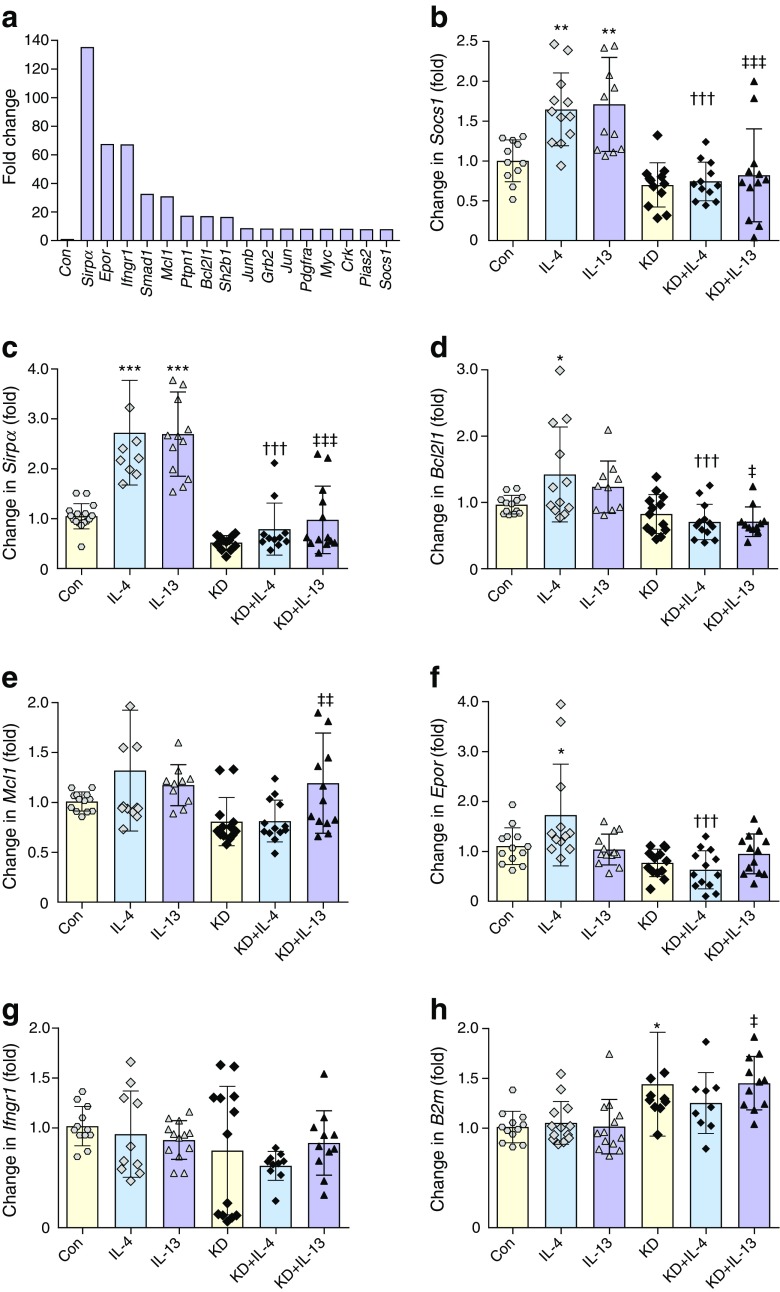
IL-13 stimulation of INS-1 cells induces the upregulation of apoptotic and anti-inflammatory genes in a STAT6-dependent manner. (**a**) RNA extracted from INS-1 cells treated with or without IL-13 (20 ng/ml) for 48 h was used to generate cDNA. The samples were analysed using a JAK/STAT RT2 Profiler PCR Array to identify genes for which expression was altered by IL-13. (**b**–**h**) Specific primers were generated for qRT-PCR analysis of a selection of genes upregulated in the array: *Socs1* (**b**), *Sirpα* (**c**), *Bcl2l1* (**d**), *Mcl1* (**e**), *Epor* (**f**), *Ifngr1* (**g**) and *B2m* (**h**). qRT-PCR was performed on cDNA generated from unmodified INS-1 cells, from cells in which STAT6 was selectively knocked down or from cells treated with IL-4 or IL-13 for 48 h. Gene expression (calculated as fold change) was measured after normalising the data to *Yy1* and *Hprt1* genes. In (**a**), data represent fold change vs control (without IL-13); (**b**–**h**), data represent fold change vs relevant control treatment and are mean values from three independent experiments ± SEM. *p<0.05, **p<0.01 and ***p<0.001 relative to control; ^†††^*p*<0.001 relative to IL-4; ^‡^*p*<0.05, ^‡‡^ *p*<0.01, ^‡‡‡^*p*<0.001 relative to IL-13. Con, control; KD, STAT6 knockdown

**Fig. 4:**
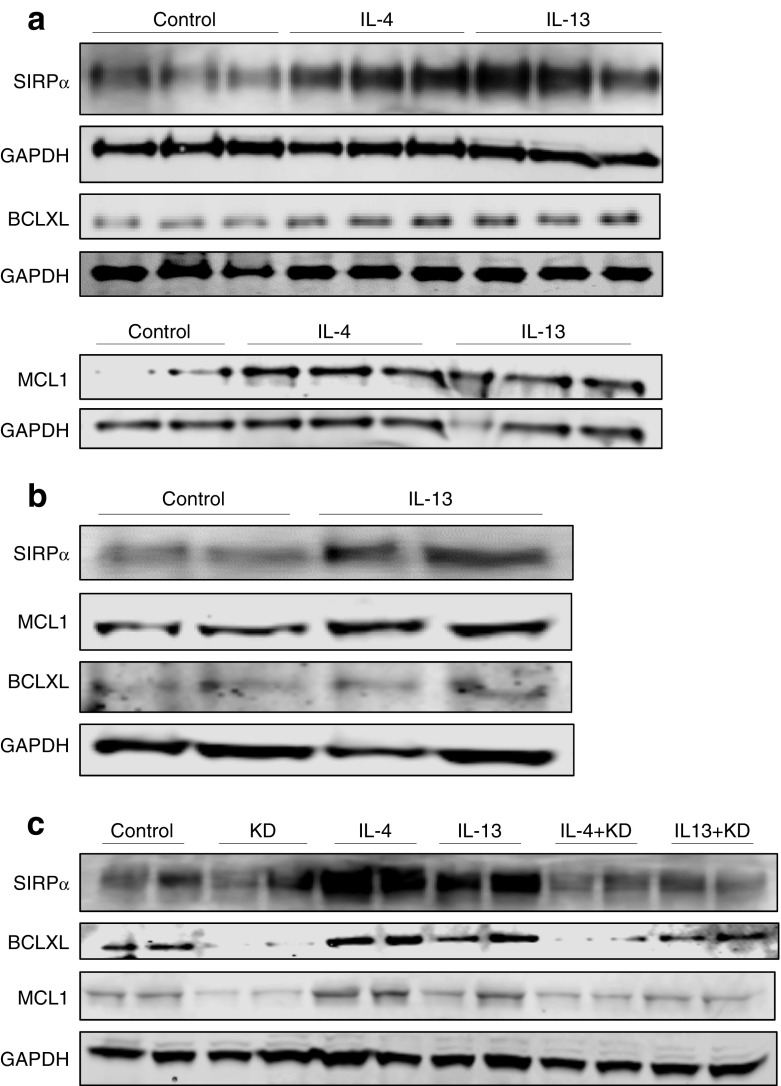
SIRPα, BCLXL and MCL1 are upregulated by IL-13 via STAT6. INS-1E cells (**a**, **c**), or EndoC-βH1 cells (**b**) were stimulated with IL-13 (20 ng/ml) (**a**–**c**) and/or IL-4 (20 ng/ml) (**a**, **c**) for 48 h, and cell lysates collected. In some experiments, IL-13 or IL-4 was employed after STAT6 knockdown (KD). Western blotting analysis was performed and membranes probed using antisera against SIRPα, BCLXL and MCL1. GAPDH was used as a loading control. These data are representative of three independent experiments

Given that the cytoprotective response to IL-13, mediated by STAT6, was most evident following a period of pre-treatment in INS-1E cells, we next examined whether it was accompanied by changes in gene expression. To our knowledge, the transcriptional response to STAT6 activation has not been characterised extensively in beta cells. Therefore, RNA was extracted from INS-1E cells treated with IL-13 for 48 h and cDNA was synthesised for analysis using a targeted PCR array. A range of genes were upregulated robustly under these conditions, with the largest increases observed in the levels of *Sirpα*, *Mcl1*, *Bcl2l1*, *Epor*, *Smad1*, *Ptpn1*, *Socs1* and *Sh2b1* (Fig. 3a). qRT-PCR analysis confirmed that *Sirpα* and *Socs1* were significantly upregulated by IL-13 (Fig. 3b, c) whereas the increases in *Bcl2l1* and *Mcl1* did not achieve statistical significance by this method (Fig. 3d,e). Importantly, however, western blot analysis confirmed the upregulation of signal regulatory protein α (SIRPα), myeloid cell leukaemia-1 (MCL1) and B cell lymphoma-extra large (BCLXL), the gene product of *Bcl2l1*, following exposure of INS-1E cells to IL-13 (Fig. 4a, ESM Fig. 1). Exposure of cells to IL-4 increased *Socs1*, *Sirpα*, *Bcl2l1*, and *Epor* mRNA levels (Fig. 3b–d, f) and, as in the case of IL-13, this was confirmed at the protein level in cell extracts (Fig. 4a, c, ESM Fig. 1). Neither IL-13 nor IL-4 altered *Ifngr1* expression as judged by qRT-PCR analysis, despite the large increase in expression observed in the PCR array (Fig. 3g). Knockdown of STAT6 abrogated the stimulatory effects of IL-13 and IL-4 on *Sirpα*, *Socs1*and *Bcl2l1* mRNA and their corresponding gene products (Figs 3b–d, f, g and 4c). Equivalent IL-13-induced changes in SIRPα were observed in human EndoC-βH1cells (Fig. 4b). MCL-1 protein expression was elevated in response to IL-13 in human EndoC-βH1cells, although the effects on BCLXL were less marked in these cells (Fig. 4b).

### SIRPα is a novel regulator of beta cell viability

**Fig. 5:**
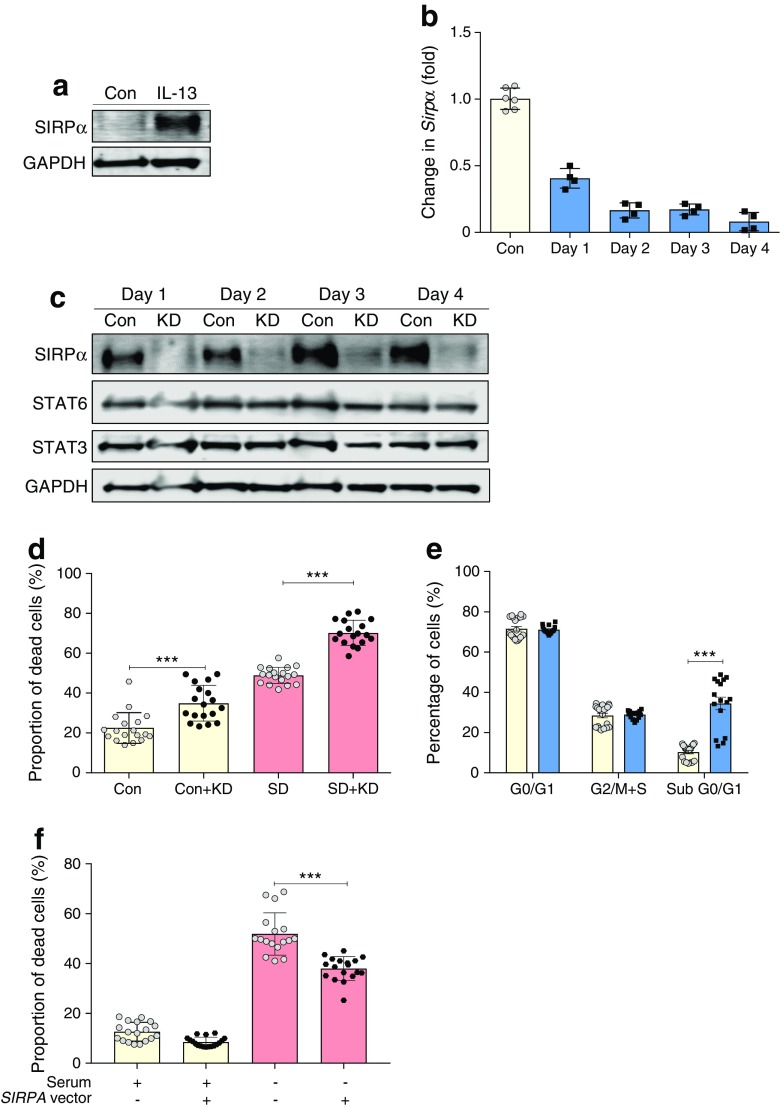
SIRPα is expressed in human islets and is a novel regulator of beta cell viability. (**a**) Western blot analysis was performed on protein lysates extracted from isolated human islets incubated under control conditions or with IL-13 (20 ng/ml) and the membranes probed with an antiserum against SIRPα. GAPDH was used as a loading control. (**b**, **c**) *Sirpα* expression was silenced in INS-1E cells over a 96 h period using siRNA. Knockdown of SIRPα (blue bars) was confirmed by (**b**) qRT-PCR measurement of *Sirpa* mRNA with data normalised using *Yy1* and *Hprt1* as housekeeping genes or (**c**) was assessed by western blotting, with membranes probed using antibodies raised against SIRPα, STAT6, STAT3 and GAPDH. (**d**, **f**) To examine the impact of SIRPα on beta cell viability, SIRPα was either (**d**) knocked down in INS-1E cells using specific siRNA molecules or (**f**) overexpressed using a *SIRPA* containing vector. In both experiments, transfection was performed 24 h prior to culture for a further 96 h under serum-deprived conditions. Cell viability was assessed by flow cytometry after this time. (**e**) Alternatively, cell cycle analysis was performed following SIRPα knockdown (blue bars). Here, cells were fixed in 95% ethanol prior to PI staining and analysis by flow cytometry. Indicated values were normalised after subtracting sub-G0/G1 cells. Data represent mean values from three independent experiments ± SEM. Representative blots are shown. ***p<0.001 as indicated. Con, control; KD, SIRPα knockdown; SD, serum-deprived condition

Among the gene products found to be upregulated by IL-13 and IL-4, SIRPα was the most unexpected. This protein is normally expressed at high levels on myeloid cells [34]. It has been studied only rarely in pancreatic beta cells, although we have noted a report implicating SIRPα (also known as Src homology 2 [SH2] domain-containing protein tyrosine phosphatase substrate 1 [SHPS-1]) in the control of insulin secretion [35]. We therefore used primary human islets and were able to verify that treatment with IL-13 resulted in a marked increase in SIRPα expression (Fig. 5a). To study the effects of SIRPα in more detail, we then used siRNA constructs to silence the protein in INS-1E cells. SIRPα expression was robustly knocked down over a period of at least 4 days by the targeted siRNA treatment, as confirmed by qRT-PCR (Fig. 5b) and western blot analysis (Fig. 5c). Importantly, knockdown of SIRPα did not alter the level of STAT6 in INS-1E cells (Fig. 5c). Surprisingly, however, under basal conditions, knockdown of SIRPα significantly enhanced beta cell death compared with cells treated with scrambled control siRNA (Fig. 5d). Analysis of the sub-G1 peak (corresponding to fragmented DNA) by flow cytometry confirmed the increase in cell death associated with depletion of SIRPα (Fig. 5e). Moreover, cell death induced by the withdrawal of serum from cells was exacerbated by the loss of SIRPα (Fig. 5d). It was then assessed whether increasing the expression of SIRPα in beta cells could induce the opposite effect, and promote cell viability. As expected, in these experiments, SIRPα over-expression significantly improved the viability of INS-1E cells cultured in the absence of serum (Fig. 5f). Taken together, these data implicate SIRPα as a novel regulator of beta cell viability whose levels are controlled in a STAT6-dependent manner.

### STAT6 is expressed in human pancreatic beta cells and is diminished in the islets of individuals with type 1 diabetes

**Fig. 6:**
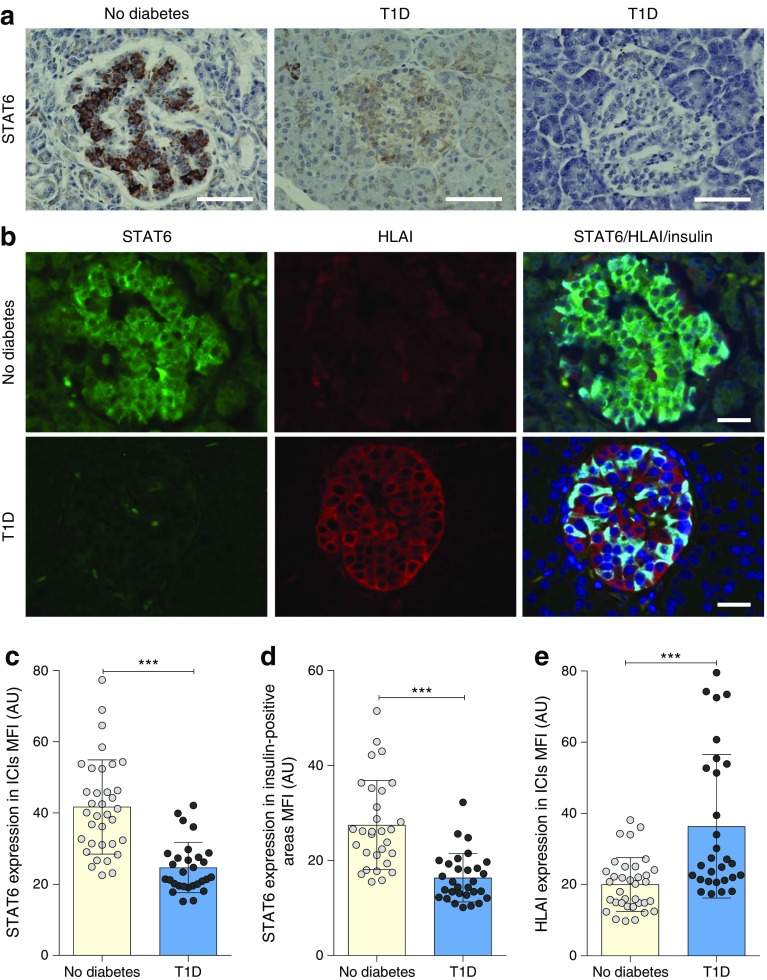
STAT6 is present in human beta cells in situ and its expression is diminished in type 1 diabetes. (**a**, **b**) Representative images of pancreas sections from individuals without diabetes and individuals with recent-onset type 1 diabetes. FFPE pancreas sections were stained for STAT6 using an immunoperoxidase approach (**a**) and co-immunofluorescence staining was employed to assess the expression of STAT6 (green), HLAI (red) and insulin (light blue) (**b**). Nuclei were stained using DAPI (**b**). Scale bars, 50 μm (**a**) or 25 μm (**b**). (**c**–**e**) To quantify these data the MFI of STAT6 (**c**, **d**) and HLAI (**e**) was determined in five ICIs from images taken at identical settings in each pancreas section. Analysis was performed either across whole islets (**c**, **e**) or only on the insulin-containing cells (**d**). Data represent means ± SEM, ***p<0.001 as indicated. AU, arbitrary units; T1D, type 1 diabetes

Immunohistochemical staining of STAT6 in human pancreas tissue from non-diabetic donors revealed a robust expression of the protein in a subset of cells in pancreatic islets, with much lower levels observed in the surrounding exocrine tissue (Fig. 6a). Co-immunofluorescence staining revealed that STAT6 is strongly co-localised with insulin, but not with glucagon, suggesting its preferential expression in beta cells (ESM Fig. 2).

To determine whether STAT6 expression is altered in diabetes, a series of six pancreas samples from individuals with recent-onset type 1 diabetes and from six healthy control donors with similar age and sex profiles were used. As expected, STAT6 was expressed robustly within the islets of control individuals but it was noticeably reduced in the insulin-containing islets (ICIs) of people with type 1 diabetes (Fig. 6a–c). To quantify these changes in expression, four or five ICIs from each individual were imaged using identical microscope and camera settings. Measurements of the MFI of the anti-STAT6 immunolabelling confirmed the downregulation of STAT6 in ICIs in type 1 diabetes (Fig. 6c, d). As previously reported, HLA class I (HLAI) expression was significantly elevated in ICIs with depleted STAT6 expression from subjects with type 1 diabetes (Fig. 6e). Since the EADB cohort is primarily an archival collection of post-mortem samples, we verified these data with samples from the Network for Pancreatic Organ Donors with Diabetes (nPOD) collection of organ donor pancreases. Immunofluorescence analysis revealed a similar pattern, with STAT6 robustly expressed in ICIs from healthy individuals but diminished in the islets of individuals with type 1 diabetes (ESM Fig. 3).

**Fig. 7:**
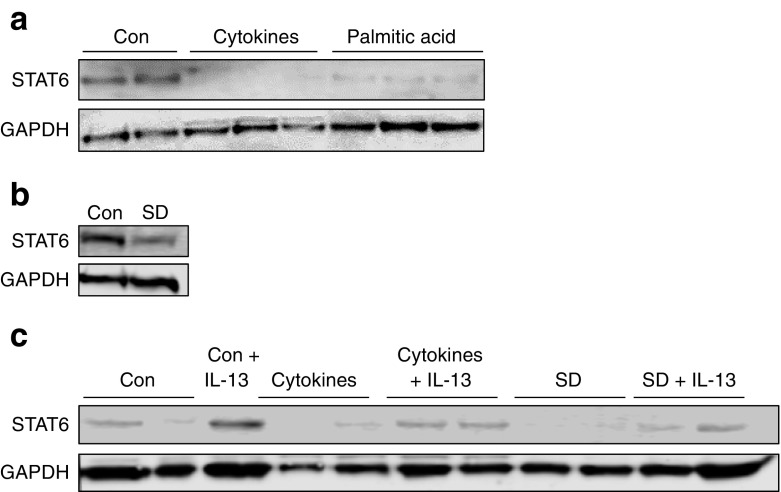
STAT6 levels are diminished in cultured INS-1E cells exposed to cytotoxic stimuli and this response is attenuated by IL-13. (**a**, **b**) INS-1E cells were cultured in the presence or absence of a proinflammatory cytokine cocktail (20 ng/ml of IL-1β, TNFα, IFNγ and IL-6) or 250 μmol/l palmitate for 48 h (**a**) or in serum-deprived (SD) media for 96 h (**b**). (**c**) Alternatively, cells were cultured under these same conditions but in the presence of IL-13. Cell lysates were collected and western blotting performed using antiserum recognising STAT6 (or GAPDH as a loading control). Data are representative of three independent experiments. Con, control

To investigate this phenomenon further, we considered the possibility that changes in the islet milieu associated with the development of diabetes might be involved in promoting the loss of STAT6. Accordingly, we used INS-1E cells which, like human islets, express high levels of STAT6 when cultured under control conditions. However, western blot analysis revealed that when these cells were treated with proinflammatory cytokines (IL1β, IFNγ, TNFα and IL-6) for 48 h or were exposed to either 250 μmol/l palmitate or conditions of serum withdrawal, STAT6 was significantly depleted (Fig. 7a, b). Interestingly, pre-treatment of the cells with IL-13 for 48 h led to an increase in STAT6 expression relative to control conditions (Fig. 7c), suggesting a feed-forward upregulation of STAT6 in cells exposed to the anti-inflammatory cytokine. Moreover, addition of IL-13 resulted in partial preservation of STAT6 expression in cells treated with the proinflammatory cytokine cocktail (Fig. 7c).

## Discussion

We show that activation of STAT6 plays an important role in maintaining the viability of pancreatic beta cells by promoting the transcription of a variety of anti-apoptotic target genes. Moreover, we reveal that the expression of STAT6 is significantly diminished in the beta cells of individuals with type 1 diabetes and propose that this is likely to enhance their susceptibility to the actions of proinflammatory cytokines during disease progression. We further show that depletion of STAT6 occurs in vitro when islet cells are exposed to proinflammatory cytokines or the saturated fatty acid palmitate (Fig. 7), suggesting that its loss may be consequent to the development of beta cell stress. Hence, our studies place STAT6 as a central component of the regulatory network controlling beta cell viability.

These conclusions arise from an analysis of the signalling pathways activated by two anti-inflammatory cytokines, IL-4 and IL-13, which culminate in STAT6 activation in beta cells. We confirm that both cytokines can protect beta cells against the cytotoxic effects of serum deprivation (used as a surrogate for growth factor withdrawal) and the presence of either proinflammatory cytokines [20–22, 25] or palmitate (Fig. 2), thereby suggesting that they are likely to be important in the context of the inflammatory milieu associated with diabetes. Although their precise origins during islet inflammation are uncertain, IL-13 and/or IL-4 may be released by specific subsets of immune cells recruited to islets during the process of insulitis. Additionally, the islet cells themselves are a potential source since IL-13 gene expression has been detected in human islets [36]. Irrespective of the endogenous sources, however, the cytoprotective effects achieved upon exogenous addition of IL-13 or IL-4 to beta cells were prevented by knockdown of STAT6, consistent with the view that a functional STAT6 pathway is required to mediate protection.

This conclusion differs from that reached recently by Rutti et al [21], who argued that the phosphatidylinositol 3-kinase (PI-3K)–Akt pathway may be of specific importance in mediating the cytoprotective effects of IL-13 in primary human beta cells. In considering these differences, we accept that it is entirely possible that multiple pathways are involved in the response but also note that the PI-3K inhibitor, wortmannin, failed to influence the cytoprotective response to IL-13 in rodent beta cells [20].

When investigating the time course over which IL-13 exerts its effects in beta cells, it was observed that tyrosine phosphorylation of STAT6 occurs as an early event but that full cytoprotection required a much longer period of incubation with IL-13. This is consistent with the accepted model in which phosphorylation of STAT6 is followed by its translocation to the nucleus and the subsequent transcription of specific target genes [27]. Accordingly, we sought to identify potential candidate genes and noted that, among a range of target molecules, two well-known anti-apoptotic genes, *MCL1* and *BCL2L1*, were markedly increased under conditions of STAT6 activation. This was confirmed at the protein level, thereby placing these gene products as being of potential importance in mediating the cytoprotective response to cytokines acting via STAT6. Both proteins are well described as anti-apoptotic molecules in the context of the beta cell [37–40] and the present data demonstrating a loss of STAT6 from beta cells in type 1 diabetes implies that anti-apoptotic responses could be downregulated under these conditions. Consistent with this, we have shown previously that MCL-1 levels are reduced in certain beta cells in the islets of people with type 1 diabetes [41] and it is worth noting that MCL-1 was also downregulated following the treatment of INS-1E cells with proinflammatory cytokines [42]. The current results imply that these effects might be contingent on STAT6 depletion.

Despite the alterations in *MCL1* and *BCL2L1*, the gene which was most robustly increased in beta cells or human islets treated with IL-13 was *SIRPα*. This gene encodes a protein, SIRPα (also known as SHPS-1), which is widely understood to function as a regulator of immune responses [43] but which has received relatively little attention in beta cells. An earlier report has implicated SIRPα in the control of insulin secretion [35] but we are not aware of any previous evidence implicating SIRPα in the control of beta cell viability or of any strong evidence for *SIRPα* being a target gene for STAT6. Thus, it was important to consider the functional role of SIRPα more fully and, accordingly, interference RNA approaches were employed to deplete the expression of the molecule in beta cells. These studies revealed that knockdown of SIRPα led to a loss of the cytoprotective actions of IL-4 and IL-13 and that depletion of SIRPα also caused a net reduction in beta cell viability under non-stimulating conditions. On this basis, we propose that SIRPα may function as a previously unrecognised regulator of beta cell viability and that increases in SIRPα, mediated by activation of STAT6, represent one important component of the downstream effector pathway by which IL-13 and/or IL-4 promote beta cell cytoprotection. The present work does not reveal the molecular pathways by which SIRPα achieves these effects but we note that beta cells also express abundant levels of its cognate binding partner, CD47 (K. A. Leslie, M. A. Russell and N. G. Morgan [principal investigator]; unpublished observations), implying that a functional signalling complex might be formed in these cells.

Interestingly, despite the loss of viability seen in the absence of a cytotoxic stimulus when SIRPα was knocked down, a similar effect was not observed upon depletion of STAT6. Rather, under these conditions, the basal viability of the cells was unaffected. These results suggest that STAT6 is unlikely to be involved in driving the constitutive expression of SIRPα in beta cells but imply that it is required to mediate the increase in expression seen upon exposure of cells to IL-13 or IL-4. Given that this then enhances the propensity of the cells to resist the effects of cytotoxic insults, these results suggest that decreases in STAT6 and SIRPα could contribute to beta cell demise during the progression of type 1 diabetes in susceptible individuals.

## Electronic supplementary material

ESM 1 (PDF 348 kb)

## Data Availability

Data are available from the authors on reasonable request.

## Abbreviations

BCLXL: B cell lymphoma-extra large
EADB: Exeter Archival Diabetes Biobank
FFPE: Formalin-fixed paraffin embedded
GAPDH: Glyceraldehyde 3-phosphate dehydrogenase
HLAI: HLA class I
ICI: Insulin-containing islets
JAK: Janus kinase
MCL-1: Myeloid cell leukaemia-1c
MFI: Mean fluorescence intensity
nPOD: Network for Pancreatic Organ Donors with Diabetes
PI: Propidium iodide
PI-3 K: Phosphatidylinositol 3-kinase
siRNA: Small interference RNA
SIRPα: Signal regulatory protein-α
STAT6: Signal transducer and activator of transcription 6
