# Supplementary material for: The transcription factor STAT6 plays a critical role in promoting beta cell viability and is depleted in islets of individuals with type 1 diabetes
Source: Diabetologia. 2018 Oct 18;62(1):87–98. doi: 10.1007/s00125-018-4750-8 (PMC6290857; doi:10.1007/s00125-018-4750-8)
Supplement: Supplementary file 1 — (PDF 348 kb) [file 125_2018_4750_MOESM1_ESM.pdf]

ESM Table 1: EASD and nPOD patient details.

Case ID	Group	Source	Donor Status	Sex	Age (years)	Duration
**485 88**	No diabetes control	UK Pancreas Biobank	Autopsy	F	2	
**146 66**	No diabetes control	UK Pancreas Biobank	Autopsy	F	18	
**191 67**	No diabetes control	UK Pancreas Biobank	Autopsy	M	25	
**330 71**	No diabetes control	UK Pancreas Biobank	Autopsy	M	47	
**540 91**	No diabetes control	UK Pancreas Biobank	Autopsy	M	11	
**21 89**	No diabetes control	UK Pancreas Biobank	Autopsy	F	4	
**6013**	No diabetes control	nPOD	Organ Donor	M	65	
**6047**	No diabetes control	nPOD	Organ Donor	M	7	
**6095**	No diabetes control	nPOD	Organ Donor	M	40	
**6099**	No diabetes control	nPOD	Organ Donor	M	14	
**E560**	Type 1 diabetes	UK Type 1 diabetes Biobank	Organ Donor	F	42	1.5y
**E567**	Type 1 diabetes	UK Type 1 diabetes Biobank	Autopsy	F	11	< 1 week
**E568**	Type 1 diabetes	UK Type 1 diabetes Biobank	Autopsy	M	10	< 1 week
**E375**	Type 1 diabetes	UK Type 1 diabetes Biobank	Autopsy	F	11	1 week
**E514**	Type 1 diabetes	UK Type 1 diabetes Biobank	Autopsy	M	23	2 weeks
**E207**	Type 1 diabetes	UK Type 1 diabetes Biobank	Autopsy	F	3	2 weeks
**6209**	Type 1 diabetes	nPOD	Organ Donor	F	5	3 months
**6228**	Type 1 diabetes	nPOD	Organ Donor	M	13	0
**6243**	Type 1 diabetes	nPOD	Organ Donor	M	13	5 years
**6245**	Type 1 diabetes	nPOD	Organ Donor	M	22	7y

ESM Table 2: Antibody details and conditions of use.

Antibody	Species raised	Dilution	Catalogue number	Company
**Anti-STAT6**	Rabbit	1:1000	5397S	Cell signalling
**Anti-STAT3**	Rabbit	1:1000	#9132	Cell signalling
**Anti-SIRPA**	Rabbit	1:1000	13379S	Cell signalling
**Anti-MCL-1**	Rabbit	1:1000	94296S	Cell signalling
**Anti β-actin**	Mouse	1:25000	A5316	Sigma-Aldrich
**Anti-pSTAT6**	Rabbit	1:500	Sc-11762	Santa Cruz
**Anti-BCLxl**	Mouse	1:1000	633901	Biolegend
**Anti-GAPDH**	Mouse	1:10000	60004-1-Ig	Proteintech
**Anti-STAT6 (IHC)**	Rabbit	1:500	sc-981	Santa Cruz
**Anti-mouse 600**	Goat	1:5000	35519	Invitrogen
**Anti-Rabbit 800**	Goat	1:5000	SA5-10036	Invitrogen
**Anti-mouse-AP**	Goat	1:25000	A3562	Sigma-Aldrich
**Anti-Rabbit-AP**	Goat	1:25000	A3687	Sigma-Aldrich
**Anti-insulin**	Goat	1:360	A0564	Dako
**Anti-glucagon**	Rabbit	1:2000	ab92517	Abcam

ESM Fig.1

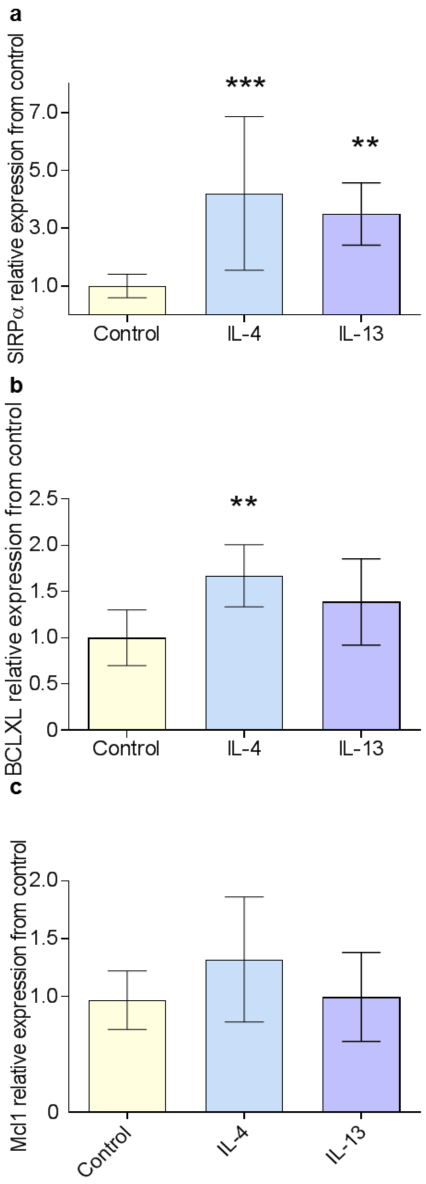

ESM Fig. 1. IL-13 and IL-4 increase the expression of SIRPα and Bcl$_x$L in INS-1E cells. Cells were treated with IL-4 (◊) or IL-13 (Δ) for 48h before extraction of protein. Samples were analysed by Western blotting using antisera to probe for SIRPα, Bcl-$_{XL}$, Mcl-1 and the loading control GAPDH (blots are presented in Fig. 4). The density of bands were calculated and normalised to GAPDH. Data are presented as fold change from untreated cells (control) ±SEM, and are from three independent experiments. **$p<0.01$, ***$p<0.001$.

ESM Fig.2

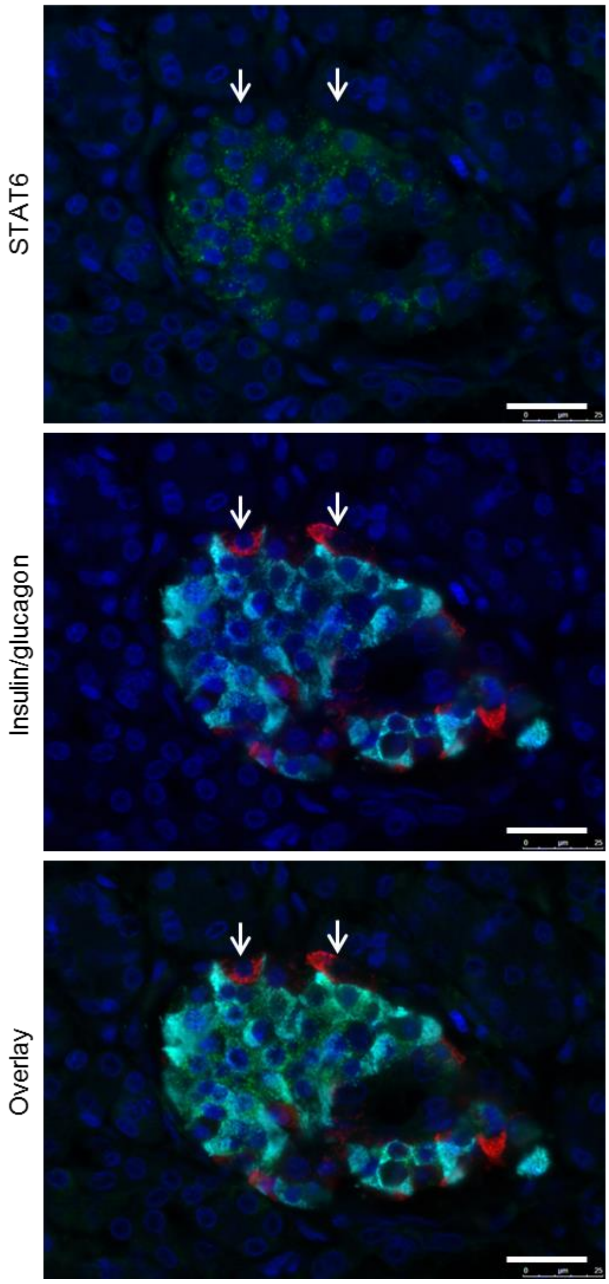

ESM Fig. 2. STAT6 is expressed in human pancreatic β-cells. A FFPE pancreas section from a control donor was stained using antibodies detecting STAT6 (green), glucagon (red) and insulin (light blue). Arrows indicated glucagon positive cells which do not have detectable STAT6 staining. Images are representative of islets on the section stained. Scale bars = 25μm.

ESM Fig.3

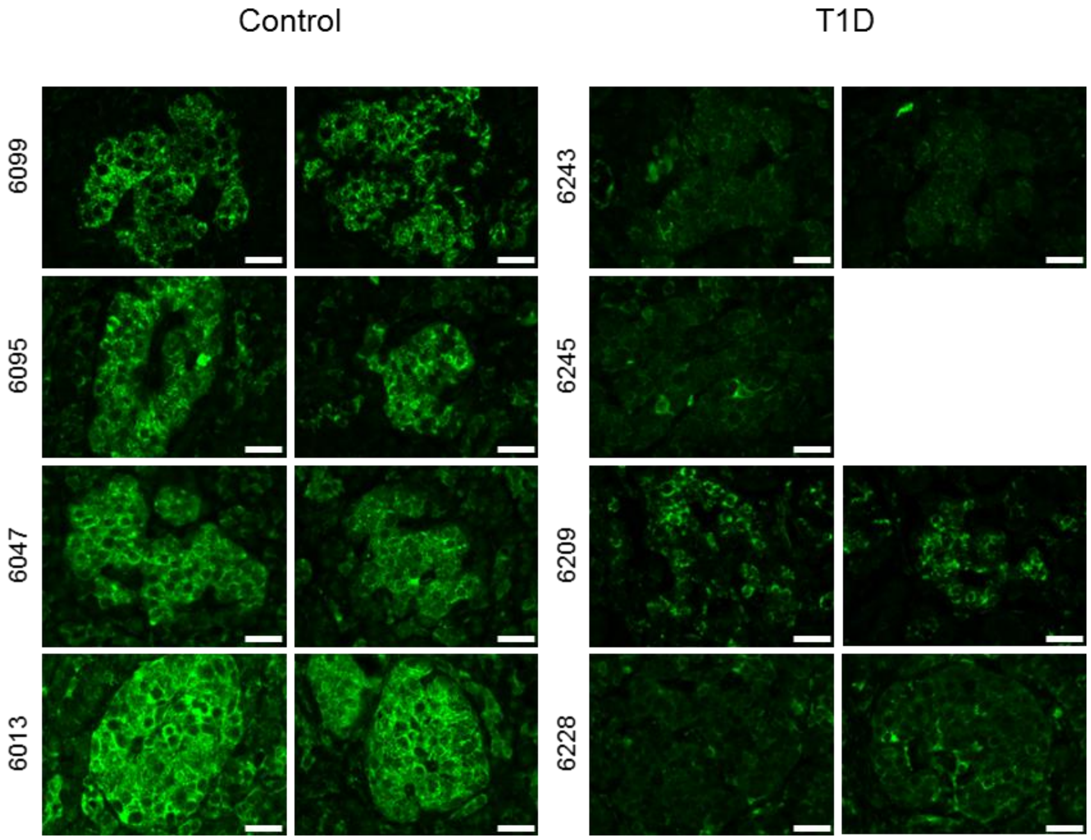

ESM Fig. 3. STAT6 expression is diminished in the pancreatic islets of patients with type 1 diabetes from the nPOD cohort. An immunofluorescence approach was employed to stain 8 pancreas sections from the nPOD collection, 4 from control donors and 4 from individuals with type 1 diabetes. Sections were stained for STAT6 (green) and insulin (not shown). Images of ICIs from each case were collected at identical settings, and two representative images from each case are presented. Only a single ICI was found on the section examined from subject 6245. Scale bars = 25μm.
